# From genetic correlations of Alzheimer’s disease to classification with artificial neural network models

**DOI:** 10.1007/s10142-023-01228-4

**Published:** 2023-09-08

**Authors:** Claudia Cava, Salvatore D’Antona, Francesca Maselli, Isabella Castiglioni, Danilo Porro

**Affiliations:** 1 Institute of Molecular Bioimaging and Physiology, National Research Council (IBFM-CNR), Via F. Cervi 93, Segrate-Milan, 20090 Milan, Italy; 2 Department of Science, Technology and Society, University School for Advanced Studies IUSS Pavia, Palazzo del Broletto, Piazza Della Vittoria 15, 27100 Pavia, Italy; 3 Department of Physics “Giuseppe Occhialini”, University of Milan-Bicocca Piazza Dell’Ateneo Nuovo, 20126 Milan, Italy; 4 NBFC, National Biodiversity Future Center, 90133 Palermo, Italy

**Keywords:** Alzheimer, Gene expression, Genetic correlation, Neural network

## Abstract

Sporadic Alzheimer’s disease (AD) is a complex neurological disorder characterized by many risk loci with potential associations with different traits and diseases. AD, characterized by a progressive loss of neuronal functions, manifests with different symptoms such as decline in memory, movement, coordination, and speech. The mechanisms underlying the onset of AD are not always fully understood, but involve a multiplicity of factors. Early diagnosis of AD plays a central role as it can offer the possibility of early treatment, which can slow disease progression. Currently, the methods of diagnosis are cognitive testing, neuroimaging, or cerebrospinal fluid analysis that can be time-consuming, expensive, invasive, and not always accurate. In the present study, we performed a genetic correlation analysis using genome-wide association statistics from a large study of AD and UK Biobank, to examine the association of AD with other human traits and disorders. In addition, since hippocampus, a part of cerebral cortex could play a central role in several traits that are associated with AD; we analyzed the gene expression profiles of hippocampus of AD patients applying 4 different artificial neural network models. We found 65 traits correlated with AD grouped into 9 clusters: medical conditions, fluid intelligence, education, anthropometric measures, employment status, activity, diet, lifestyle, and sexuality. The comparison of different 4 neural network models along with feature selection methods on 5 Alzheimer’s gene expression datasets showed that the simple basic neural network model obtains a better performance (66% of accuracy) than other more complex methods with dropout and weight regularization of the network.

## Introduction

Alzheimer’s disease (AD), the most common form of dementia with 60–70% of total cases, has an onset over 65 years of age. AD is a progressive, incurable neurodegenerative disease characterized by a gradual decline in cognition, memory, and thinking (Kumar et al. 2022).

Currently, the therapeutic approaches offer limited results in symptoms and progression of the disease, but there is not a definitive treatment (Lane et al. 2018; Brookmeyer et al. 1998). Thus, a great challenge is to develop novel methods for early detection, in order to decrease or prevent disease progression.

Sporadic cases of AD originate from complex genetic architecture that involves many risk loci with small single influences (Tesi et al. 2021). Indeed, AD-associated SNPs were common with other medical conditions and human traits (Tesi et al. 2021). Genetic correlation analysis based on phenome-wide screening generates novel hypotheses related to risk conditions and comorbid events of AD (Liu and Crawford 2022). Rapid rising of high-throughput technologies (e.g., microarray and next generation sequencing) over the past years has resulted in a significant recent increase of novel computational methods of many diseases including AD (van IJzendoorn et al. 2019).

Genome-wide association studies (GWAS) are essential tools to address this complexity and opening up new therapeutic challenges. Previous GWAS in AD demonstrated the association between immune system and lipid metabolism and identified several genes and genetic variants related to lipid metabolism (Baloni et al. 2022; Kunkle et al. 2019). In addition, recent studies demonstrated that cardiovascular and life style factors could increase the risk of AD development as well as diabetes, obesity, and hypertension (Broce et al. 2019; Desikan et al. 2015). Adewuyi et al. showed an association between gut and brain, suggesting a potential genetic susceptibility of gastrointestinal disorders with AD’s risk (Adewuyi et al. 2022). Another group with AD genetically associated traits is related to the dietary habits (Squitti et al. 2014). For example, a lower incidence of AD has been reported in subjects following a Mediterranean diet (Gardener et al. 2012). The lack of micronutrients in the diet such as vitamins B1, C, and folate has been related to cognitive decline in elderly people (Solfrizzi et al. 2011). However, genetic correlation is not a diagnostic tool, but a method to establish the genetic similarity between complex traits (van IJzendoorn et al. 2019).

Most machine learning algorithms proposed for AD classification are based on phenotypic data such as imaging, and few studies use genetic data (Lee et al. 2018). Since 2012, deep learning, a branch of machine learning, has been shown good performance in several areas outside biological problems (Abiodun et al. 2018). Lately, with this assumption, several studies have demonstrated the potential of deep learning to address also biological questions as diagnostic tools (Zhu et al. 2020a; Rukhsar et al. 2022). Deep learning indicates machine learning algorithms that are composed of deep neural networks. Different studies are based on the biological application of neural networks and few studies are focused on how the network architectures could improve the performance of models (Bellot et al. 2018; Yu et al. 2019; Wilentzik Müller and Gat-Viks 2020). There are different challenges in obtaining the optimal model of neuronal network in a classification problem (Rukhsar et al. 2022). Mainly, the performance of neural network models can be influenced by the amount of data that could generate overfitting problems (Esteva et al. 2019). To resolve this problem, researchers have developed several techniques such as regularization methods, dropout, class balanced, and feature selection (Esteva et al. 2019). However, there is not a best general method because it is difficult to obtain the performance of each network architecture using a same dataset (Nusrat and Jang 2018; Moolayil 2019). In addition, unlike imaging or text data, classifiers based on neural networks are still novel in gene expression analysis (Hanczar et al. 2022).

In this work, using genome-wide associations statistics from public datasets, we explored a genetic correlation between AD and many other human traits. In addition, we considered and compared different methods to reduce the overfitting using gene expression profiles of Alzheimer patients derived by 5 published datasets. The dimensionality of the gene expression profiles was reduced with principal component analysis (PCA) as it transforms the features into a lower dimensional space considering the relationships between the features. Furthermore, after a procedure to avoid unbalanced classes, we evaluated 5 network models considering the performance of the classifier with accuracy, sensitivity, and specificity.

The aims of our study are (i) to identify the mechanisms of AD genetic liability that could be connected to different human traits, (ii) to explore artificial neural network models for AD diagnosis, (iii) comparison of different models based on artificial neural network using gene expression profiles of AD patients. In particular, the present findings could (i) suggest future studies to assess the impact of several traits with AD risk and (ii) open new potential frontiers in the study of AD.

## Materials and methods

### Genome-wide association studies

GWAS summary statistics of AD were downloaded from GWAS Atlas (Jansen et al. 2019). The dataset consists of European cohort and have participants of both sexes. We used the summary statistics of 71,880 AD cases and 383,378 controls (Jansen et al. 2019).

We also considered genome-wide association statistics of other phenotypes and diseases, derived from the UK Biobank (UKB) (Bycroft et al. 2018). This dataset was downloaded from (http://www.nealelab.is/uk-biobank/, accessed on 4 February 2022).

The UK Biobank enrolled approximately 500,000 participants aged 40–69 years, of both sexes from the UK (Bycroft et al. 2018). UKB participants were analyzed for a wide range of phenotypic information such as diet, educational status, cognitive function, social activities, health status, and other phenotypes.

Data quality control was performed separately for each data set (AD and UKB).

In particular, we calculated SNP-heritability for AD and UKB phenotypes and considered for further analyses only phenotypes with SNP-heritability z > 4.

In addition, AD and UKB genome-wide association statistics were processed by removing SNP with a minor allele frequency (MAF) < 1%. More detailed descriptions of quality control step are available at https://github.com/Nealelab/UK_Biobank_GWAS.

### Genetic correlation analysis

We estimated the genetic correlation analysis between the AD phenotypes and the other phenotypes included in UKB. To perform the genetic correlation, we used the package linkage disequilibrium score regression (LDSC) (Accessed on May 2022; LD Score: https://github.com/bulik/ldsc) (). This is a method that performs the linkage disequilibrium (LD) mechanism to calculate the distribution of effect sizes for each SNP, thus assigning the score and the type of correlation between phenotypes.

We used SNPs present in the HapMap 3 reference panel, and as reference data, the individuals of European ancestry from the 1000 Genomes Project. We performed the genetic correlation analysis between AD phenotype with UKB traits with SNP-based heritability z score > 4, in line with the guidelines of the LDSC developers (). We considered statistically significant genetic correlations as those that had an FDR less than 0.05.

### Gene expression data

Five publicly available datasets of gene expression profiles of Alzheimer patients (GSE1297, GSE5281, GSE36980, GSE29378, and GSE48350) were downloaded from the Gene Expression Omnibus (GEO). These datasets contain the gene expression profiles of hippocampus of Alzheimer patients as this brain area is involved in the early stages of disease (Quarato et al. 2022). We considered hippocampus because we suppose that this part of brain plays a fundamental role in different traits associated with AD. Table 1 shows the number of samples for Alzheimer patients and controls of each dataset.

**Table 1:** Number of samples for each class

GEO dataset	Alzheimer	Control
GSE1297	22	9
GSE5281	10	13
GSE36980	8	10
GSE29378	31	32
GSE48350	19	43

### Training and testing sets

We split each GEO dataset into two sets: training and testing sets. Neural network was trained using the training set and the testing set to test the model: 70% of the original dataset for the training and 30% for the testing.

To avoid unbalanced datasets, namely a number of samples in a class (e.g., Alzheimer) is greater than another class (e.g., control), we performed a random oversampling. This approach balances the minority class with majority class.

In addition, we standardized each dataset separately converting the data distribution per feature to a normal distribution.

### Feature selection

The presence of unrelated features in the dataset can decrease the accuracy of the models. During the feature selection step, we selected a subset of features that contribute to reduce overfitting (Moolayil 2019). PCA was used to decrease the dimensionality of datasets and to identify the key components on the standardized training data (Moolayil 2019). The number of basic components according to the training data was defined considering 95% of the variance of the data. We considered the same components in both the training and testing data.

### Neural network models

Similar to other machine learning methods, neural network model is composed of (i) the training step where the parameters of the network are estimated from a given training dataset and (ii) the testing step that applies the trained network to predict the classes of new input data.

The neurons in our models are organized in 4 layers where all nodes in a specific layer must be connected to all the nodes in the next layer.

In all models, the four layers were defined as follows: the input (first) layer consists of a number of neurons equal to the number of features (i.e., key components derived by PCA). The first hidden layer characterized by 17 neurons and the second hidden layer of 8 neurons. The output layer returns the predicted class.

Each neuron calculates a weighted sum of its inputs and applies an activation function. We used a model rectified linear unit (ReLU) at each node of the network for all models (Glorot et al. 2011). It is the most common activation function used and it generates 0 as output for negative inputs, following the formula:

$$\mathrm{f}\left(\mathrm{x}\right)=\mathrm{max}\left(0,\mathrm{x}\right)$$

A sigmoid activation function is used for the output layer to identify the class to be predicted for all model:

$$\mathrm{sigmoid}\left(\mathrm{x}\right)=1/\left(1+\mathrm{exp}\left(-\mathrm{x}\right)\right)$$

where x is a feature vector.

The Adam stochastic gradient descent optimization is used as optimizer algorithm in all our models to train the network (Kingma and Ba 2014). It assigns the parameters that decreases the loss function. Gradient descent uses the first derivate of the activation function to modify the parameters of the model. Specifically, Adam changes the weights of the model in the training set iteratively to maximize a particular class (Kingma and Ba 2014).

Table 2 shows the parameters considered for all 4 models. We evaluated the parameters as setted in Izadkhah 2022.

**Table 2:** Description of parameters used in artificial neural network (ANN) models

Model	Parameters
ANN	Number of hidden layers = 2
	Batch size = 8
	Epochs = 200
	Optimizer = adam
	Hidden layers activation function = relu
	Output layer activation function = sigmoid

We tested 4 different neural network architectures for the binary classification problem which differ in loss function, metrics, dropout, and weight regularization.

The loss functions to minimize during the training that we used are binary cross-entropy or mean squared logarithmic error. This function is used to evaluate the classifier through the model error and to quantify how the model fits (Rengasamy et al. 2020).

It is a common strategy to reduce the overfitting of neural network to add dropout or introduce a penalty (weight regularization).

A dropout can be used to decrease the overfitting of the model. The dropout consists in removing a random subset of nodes (Srivastava et al. 2014).

Table 3 shows the 4 different models of neural networks used.

**Table 3:** Description of neural network models

Model	Loss function		Metric		Dropout	Weight reg
	Binary cross-entropy	Mean squared logarithmic error	Accuracy	Binary accuracy		
1	X			X		
2		X	X			
3		X	X		X	
4		X	X			X

Summarizing, the different 4 models are organized as follows:

- First model: The first model consists of binary cross-entropy as loss function, adam as optimization algorithm, and the binary accuracy as metric. Binary cross-entropy determines the cross-entropy loss between the predicted classes and the true labels.
- Second model: The second model consists of mean squared logarithmic error as loss function, adam as optimization algorithm, and the accuracy as metric. Mean squared logarithmic error is calculated between the true classes and predicted classes.
- Third model: The third model consists of mean squared logarithmic error as loss function, adam as optimization algorithm, and as metric the accuracy. Dropout is applied between the second and third layer to reduce overfitting and the dropout rate is set to 0.5. Dropout consists of a random selection of a small number of nodes instead of all nodes changing by regularly the nodes in the training process (Kingma and Ba 2014).
- Fourth model: The fourth model consists of mean squared logarithmic error as loss function, adam as optimization algorithm, and as metric the accuracy. Weight regularization is used to reduce overfitting and regularization hyper-parameter value is set to 0.001. Weight regularization is a method to reduce the overfitting by regulating the weight distribution adding a regularization expression to the cost function (loss function) (Maki 2019). Weight regularization to reduce the error is based on criterion in our study: it adds “summed squared weights” as penalty term to the loss function (Maki 2019).

In all models, we introduced an “early stopping function” presents in the package Keras (https://keras.io/callbacks/#earlystopping) that regularly checks loss values of testing data and stops the training process when there is not a significant improvement in the loss values of the testing data. The quantification of acceptable improvement is set to 0.005 and if there are not improvement of the loss values in at the last 5 interactions the training process will terminate. To reduce the time and memory activity, the model was trained with a batch size = 8 and run for a maximum of 200 epochs.

Finally, we compared the performance of the 4 models (sensitivity, specificity, and accuracy) in the testing set for each GEO dataset. It must be noted that neural networks are based on stochastic algorithms and so, the performance on the same data with same model can slightly differ. In order to obtain more realistic results, we calculated the average sensitivity, average specificity, and average accuracy running 10 times the same model.

The neural network model code was implemented in Python using the keras package (version 2.10).

## Results

### Genetic correlation

After quality control step, the number of SNPs in GWAS of AD is reduced from 13,367,299 to 9,736,043 SNPs. Out of 4000 phenotypes of UKB, only 957 passed the quality control.

Genetic correlation analysis can demonstrate if AD is influenced by external factors. We found 65 traits correlated with AD (Table 4).

**Table 4:** The table shows genetic correlation (GC) with the respective standard error and associated FDR

Macro-groups	Disease vs phenotype	GC (sd)	FDR
Diseases and medical conditions	AD vs diseases of the digestive system	− 0.32 (0.0881)	2.61E-02
	AD vs overall health rating	− 0.27 (0.0729)	2.61E-02
	AD vs illnesses of mother: none of the above (group 1)	0.32 (0.0862)	2.61E-02
	AD vs taking other prescription medications	− 0.28 (0.0768)	2.61E-02
	AD vs symptoms signs and abnormal clinical and laboratory findings not elsewhere classified	− 0.32 (0.0958)	2.97E-02
	AD vs mood swings	− 0.22 (0.0655)	2.97E-02
	AD vs diseases of the genitourinary system	− 0.33 (0.0957)	2.97E-02
	AD vs diseases of the musculoskeletal system and connective tissue	− 0.33 (0.1009)	2.97E-02
	AD vs other serious medical condition or disability diagnosed by doctor	− 0.33 (0.0967)	2.97E-02
	AD vs frequency of tiredness or lethargy in last 2 weeks	− 0.26 (0.0767)	2.97E-02
	AD vs long standing illness disability or infirmity	− 0.30 (0.0868)	2.97E-02
	AD vs medication for pain relief constipation heartburn: none of the above	0.25 (0.0751)	3.10E-02
	AD vs pain type(s) experienced in last month: none of the above	0.25 (0.0751)	3.10E-02
	AD vs attendance or disability or mobility allowance: none of the above	0.3 (0.0906)	3.10E-02
	AD vs non-cancer illness code self-reported osteoarthritis	− 0.36 (0.1103)	3.20E-02
	AD vs any ICDMAIN event in Hilmo or causes of death	− 0.26 (0.0835)	3.91E-02
	AD vs treatment or medication code lansoprazole	− 0.36 (0.116)	4.20E-02
	AD vs wheeze or whistling in the chest in last year	− 0.25 (0.0822)	4.53E-02
	AD vs had major operations	− 0.36 (0.1189)	4.53E-02
	AD vs non-cancer illness code self-reported depression	− 0.27 (0.0905)	4.63E-02
	AD vs attendance or disability or mobility allowance: disability living allowance	− 0.24 (0.0813)	4.85E-02
Fluid intelligence	AD vs FI3 word interpolation	0.30 (0.0941)	3.10E-02
	AD vs fluid intelligence score	0.21 (0.0696)	4.53E-02
Education and qualifications	AD vs qualifications: college or university degree	0.27 (0.071)	2.61E-02
	AD vs qualifications: none of the above	− 0.27 (0.0741)	2.61E-02
	AD vs age completed full time education	0.29 (0.081)	2.97E-02
	AD vs qualifications O levels or GCSEs or equivalent	0.28 (0.0822)	2.97E-02
	AD vs qualifications A levels or AS levels or equivalent	0.22 (0.071)	3.50E-02
Anthropometric measurements	AD vs standing height	0.19 (0.0535)	2.61E-02
	AD vs father age at death	0.3 (0.0868)	2.97E-02
	AD vs forced expiratory volume in 1 s (FEV1) predicted	0.3 (0.0645)	2.97E-02
	AD vs leg fat percentage (left)	− 0.21 (0.0672)	4.40E-02
	AD vs high light scatter reticulocyte count	− 0.14 (0.0473)	4.53E-02
	AD vs leg fat percentage (right)	− 0.2 (0.0661)	4.53E-02
	AD vs forced vital capacity (FVC) best measure	0.17 (0.0566)	4.63E-02
	AD vs body mass index (BMI)	− 0.19 (0.0645)	4.63E-02
Employment status	AD vs current employment status unable to work because of sickness or disability	− 0.34 (0.1016)	2.97E-02
	AD vs current employment status in paid employment or self employed	0.43 (0.1395)	3.91E-02
Activity	AD vs types of physical activity in last 4 weeks other exercises (e.g., swimming cycling keep fit bowling)	− 0.25 (0.0743)	2.97E-02
	AD vs time spent watching television (TV)	0.29 (0.0889)	3.10E-02
	AD vs leisure or social activities sports club or gym	0.23 (0.0711)	3.20E-02
	AD vs time spent using computer	− 0.19 (0.0604)	3.64E-02
	AD vs usual walking pace	− 0.23 (0.0778)	4.53E-02
	AD vs duration screen displayed	− 0.21 (0.0702)	4.85E-02
Diet	AD vs alcohol drinker status previous	− 0.39 (0.1172)	2.97E-02
	AD vs never eat eggs dairy wheat sugar or foods or drinks containing sugar	0.22 (0.0688)	3.10E-02
	AD vs cereal-type Muesli	0.24 (0.0715)	3.10E-02
	AD vs dried fruit intake	− 0.23 (0.0777)	4.53E-02
	AD vs alcohol usually taken with meals	− 0.22 (0.0731)	4.53E-02
	AD vs salt added to food	0.23 (0.0775)	4.63E-02
	AD vs never eat eggs dairy wheat sugar I eat all of the above	0.19 (0.064)	4.85E-02
Housing and lifestyle	AD vs own or rent accommodation lived in rent from local authority local council housing association	− 0.39 (0.1094)	2.61E-02
	AD vs someone to take to doctor when needed as a child	0.48 (0.1445)	2.97E-02
	AD vs spells in hospital	− 0.34 (0.1015)	2.97E-02
	AD vs job involves heavy manual or physical work	0.26 (0.0773)	2.97E-02
	AD vs average total household income before tax	0.261 (0.0735)	2.97E-02
	AD vs job involves mainly walking or standing	0.25 (0.0772)	3.10E-02
	AD vs smoking status: never	0.19 (0.0598)	3.10E-02
	AD vs exposure to tobacco smoke at home	− 0.40 (0.1312)	4.30E-02
	AD vs smoking status current	− 0.26 (0.0847)	4.53E-02
	AD vs smoking or smokers in household	0.37 (0.1248)	4.63E-02
Sexuality	AD vs age at last live birth	0.26 (0.0829)	4.04E-02
	AD vs age at first live birth	0.3 (0.0821)	2.61E-02
	AD vs age first had sexual intercourse	0.28 (0.072)	2.61E-02
	AD vs age started oral contraceptive pill	0.33 (0.0918)	2.61E-02

Twenty-one out of sixty-five AD significant genetic correlations were associated to traits related to medical conditions. In particular, AD was negatively correlated with 17 traits. In addition, AD was positively associated with Fluid intelligence score (gc = 0.21, *p* = 4.53E-02). 6 AD genetic correlations were related to activity. In particular, it was inversely correlated with Time spent using computer (gc =  − 0.19, *p* = 3.64E-02) and positively correlated with time spent watching television (TV) (gc = 0.29, *p* = 3.10E-02). Related to education, ALS genetic correlation was positively associated with high qualification (gc = 0.27, *p* = 2.61E-02)

### Neural network models

As first aim of our work, we investigated if neural networks can be used as tool for Alzheimer diagnosis (i.e., classification Alzheimer vs control) considering 5 gene expression datasets. For each of these datasets, we explored different neural network models.

**Fig. 1:**
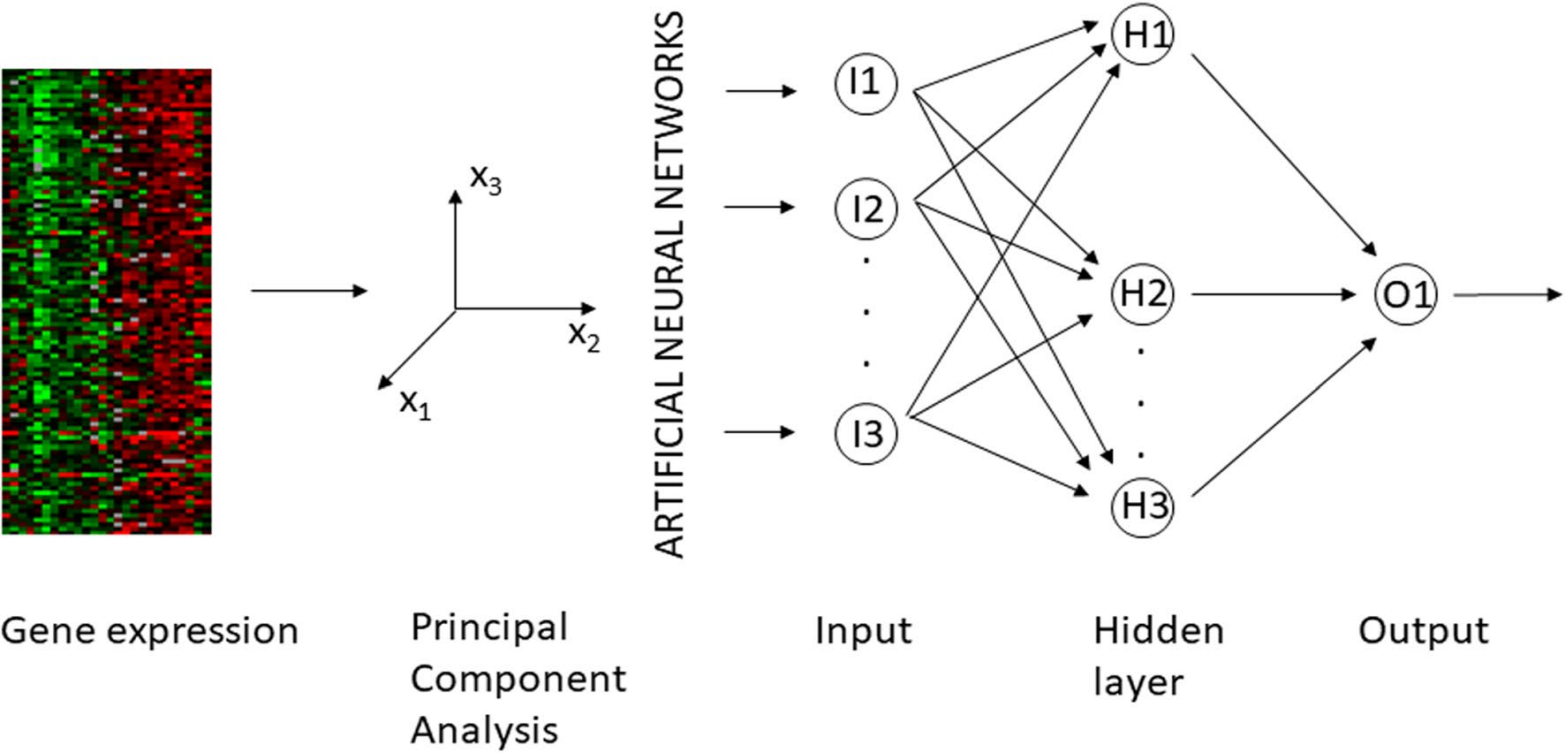
Neural network structure used in the study. The input layer is the results of principal component analysis; the output layer consists of one node describing the class of the sample

All neural network models consist of three layers. Input nodes equal to the number of input feature (i.e., key components derived by PCA). We used as hidden layer a number of 50% of input nodes. Being a binary classification, the models require an only output node. Figure 1 shows the described neural network.

We investigated 4 neural network models. As the most basic neural network structure, we examined a neural network that uses binary cross-entropy as loss function and binary accuracy as metric to evaluate the model in the training. The classification model was demonstrated to be more accurate in GSE5281 (accuracy 0.78, sensitivity 0.68, and specificity 0.88) achieving an overall average good performance in all datasets (accuracy 0.66, sensitivity 0.62, and specificity 0.712).

We then tested the classification using a model neural network based on mean squared logarithmic error as loss function and the accuracy as metric. The average performance of all GEO datasets showed a dramatic decrease: accuracy 0.546, sensitivity 0.524, and specificity 0.566. Similar results were obtained with the third model that used dropout: accuracy 0.554, sensitivity 0.492, and specificity 0.628.

A slight improvement was achieved with the weight regularization in the fourth model: accuracy 0.582, sensitivity 0.6, and specificity 0.566.

Table 5 shows the performance of the classifier for each GEO dataset.

**Table 5:** Performance (accuracy, sensitivity, and specificity with standard deviation) for each neural network model and for each GEO dataset

Model	GEO dataset	Accuracy	Sensitivity	Specificity
1	GSE1297	0.54 ± 0.19	0.6 ± 0.27	0.48 ± 0.35
	GSE5281	0.78 ± 0.23	0.68 ± 0.33	0.88 ± 0.19
	GSE36980	0.78 ± 0.22	0.63 ± 0.37	0.93 ± 0.14
	GSE29378	0.58 ± 0.16	0.65 ± 0.24	0.52 ± 018
	GSE48350	0.65 ± 0.14	0.54 ± 0.24	0.75 ± 0.11
	Mean ± SD	0.66 ± 0.188	0.62 ± 0.29	0.712 ± 0.194
2	GSE1297	0.42 ± 0.16	0.36 ± 0.26	0.48 ± 0.35
	GSE5281	0.59 ± 0.28	0.64 ± 0.36	0.54 ± 0.44
	GSE36980	0.55 ± 0.26	0.63 ± 0.43	0.47 ± 0.48
	GSE29378	0.57 ± 0.12	0.53 ± 0.37	0.62 ± 0.37
	GSE48350	0.6 ± 0.15	0.46 ± 0.39	0.72 ± 0.27
	Mean ± SD	0.546 ± 0.194	0.524 ± 0.362	0.566 ± 0.382
3	GSE1297	0.44 ± 0.21	0.31 ± 0.31	0.58 ± 0.47
	GSE5281	0.67 ± 0.22	0.5 ± 0.43	0.84 ± 0.35
	GSE36980	0.53 ± 0.23	0.69 ± 0.4	0.37 ± 0.46
	GSE29378	0.56 ± 0.1	0.47 ± 0.28	0.65 ± 0.32
	GSE48350	0.57 ± 0.1	0.49 ± 0.36	0.7 ± 0.28
	Mean ± SD	0.554 ± 0.172	0.492 ± 0.356	0.628 ± 0.376
4	GSE1297	0.56 ± 0.18	0.55 ± 0.36	0.58 ± 0.41
	GSE5281	0.62 ± 0.26	0.64 ± 0.42	0.6 ± 0.46
	GSE36980	0.58 ± 0.29	0.66 ± 0.35	0.5 ± 0.48
	GSE29378	0.53 ± 0.08	0.58 ± 0.3	0.47 ± 0.23
	GSE48350	0.62 ± 0.17	0.57 ± 0.3	0.68 ± 0.2
	Mean ± SD	0.582 ± 0.98	0.6 ± 0.346	0.566 ± 0.356

**Fig. 2:**
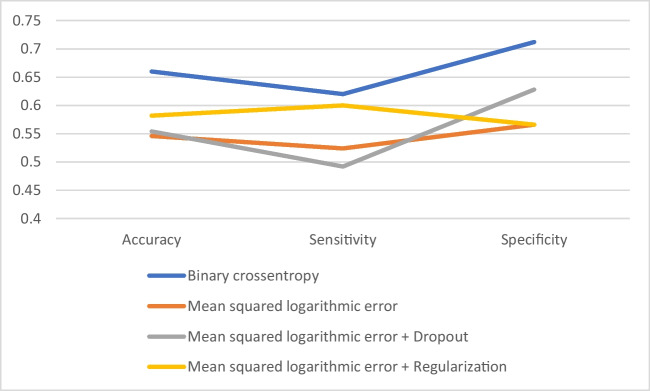
Comparison of performance for each neural network model

Overall, the best performances were achieved with the first and fourth model (Fig. 2).

## Discussion

Sporadic AD is the most common form of dementia. It is due to the effects of many risk loci with small single consequences. In the present study, we performed a genetic correlation analysis between genome-wide association statistics of AD derived by GWAS Atlas and human traits from UK Biobank.

We observed that AD was mainly associated with fluid intelligence score, medical conditions, diet, and activities. Regarding the diet, AD is positively associated with cereal and salt intake and inversely correlated with dried fruit and alcohol intake. Further studies should be performed to understand the potential beneficial effect of alcohol consumption and negative effect of salt intake.

Another macro-area with multiple AD genetically correlated phenotypes is anthropometric measurements: positively associated with standing height and inversely correlated with leg fat percentage (left), high light scatter reticulocyte count, leg fat percentage (right), and body mass index (BMI).

As hippocampus, a part of cerebral cortex, plays a central role in several traits that we found to be associated with AD, we applied to gene expression profiles of hippocampus of AD patients and artificial neural models.

Regarding the development of diagnostic tools for AD, we explored the role of artificial neural network based on gene expression of hippocampus of Alzheimer patients.

Artificial neural network, an emergent field of machine learning, is a computational model involving interconnected nodes inspired by neurons in the human brain to solve complex problems. It uses one or more hidden layers, an activation function and hyper-parameters to elaborate the input and generate the output.

Recent studies in bioinformatics have proposed the use of neural networks in molecular classification of diseases by gene expression and multi-omics data (Qiu et al. 2022; Shao et al. 2021). Many studies were focused on the comparison between artificial neuronal network and other machine learning methods, demonstrating that artificial neural networks are more flexible and work on different types of data (e.g., discrete or continuous data) (Esteva et al. 2019; Biganzoli et al. 1998; Zhu et al. 2020b).

However, few studies have been performed to evaluate different procedures to avoid overfitting and improve the performance of the artificial neuronal network considering gene expression datasets (Hanczar et al. 2022; Zhu et al. 2020b; Chen et al. 2016). This could be explained by the great number of hyper-parameters to test.

Our study compared 4 neural network models applied to gene expression datasets of Alzheimer, showing that the simple basic neural network model achieves a better performance than other more complex methods with dropout and weight regularization (accuracy 0.66, sensitivity 0.62, and specificity 0.712). However, increasing the size of the samples in the datasets, the model could further improve the performance and confirm these results. Indeed, the dataset size is a critical aspect that could influence the performance of models. Typically, larger datasets could lead to better performance and small datasets may generate overfitting (Prusa et al. 2015). Supervised machine learning methods also depend on the diversity and quality of the dataset to achieve good performances in generalization step (Leguy et al. 2021).

In line with our results, a previous study found that simple neural network models have obtained similar performance compared to other complex methods (Zhu et al. 2020b). Although the values of hyper-parameters used in this study are closely associated with our data, we can suggest the use of simple basic neural network for gene expression classification. In addition, loss function with binary cross-entropy seems to work with better performance than mean squared logarithmic error. Note, regularization methods seem to reduce the overfitting and work better than dropout procedures.

## Conclusion

In conclusion, the present study with genetic correlation analysis suggested several mechanisms of AD that could be associated with different human traits. It can be grouped into 9 clusters: medical conditions, fluid intelligence, education, anthropometric measures, employment status, activity, diet, lifestyle, and sexuality. However, correlation analysis does not necessarily imply causation, namely the cause-and-effect relationship between two variables. In order to establish causality, it is necessary to conduct further studies that can identify cause-effect relationships more reliably. In addition, further studies should be conducted to fully understand the impact of SNPs on these relationships.

Related to neural network models in our study, we compared the most suitable schemes for artificial neuronal network applied to gene expression datasets of patients with Alzheimer. Our results showed that the simple basic neural network model achieved a better performance (66% of accuracy). To our knowledge, in literature, there was not similar research, and more studies are needed to completely define standard procedures to achieve more efficient results. It could be also interesting to explore more sophisticated deep neural networks also increasing the size of the datasets.

## Data Availability

All the summary statistics used in this study are available from the United Kingdom Biobank database (http://www.nealelab.is/uk-biobank). Gene expression profiles are available from Gene Expression Omnibus (Home—GEO—NCBI (nih.gov)).
